# Mapping for the Target Sites of Ablation in Post-infarction Ventricular Tachycardia- Is Sinus Rhythm Sufficient?

**Published:** 2009-11-01

**Authors:** Narayanan Namboodiri

**Affiliations:** Sree Chitra Tirunal Institute for Medical Sciences and Technology, Trivandrum, Kerala, India

**Keywords:** ventricular tachycardia, catheter ablation

All truths are easy to understand once they are discovered; the point is to discover them - Galileo Galilei (1564 - 1642)

The long road to success with radiofrequency ablation for scar-related ventricular tachycardia (VT) has taken the electrophysiologists down many unexpected paths in the last two decades. During this period, the progress in defining the pathological circuits involved in genesis of this complex tachyarrhythmia has undoubtedly improved the ablation outcome. However, empiricism still prevails as to how optimally map the ablation targets of this arrhythmia commonly encountered in clinical practice.

It is now clear that scar-related VT is caused by macroreentrant circuits within or adjacent to the scar that forms after myocardial infarction [1,2]. Small strands of surviving myocardium or Purkinje fibres form anisotropic and slow conduction in these areas and serve as ideal substrates for re-entrant circuits. The success of VT ablation, hence, depends on identifying and ablating the critical parts of these re-entrant circuits [2,3]. In hemodynamically stable VTs, these sites can be identified by recognition of late diastolic potentials (LDP) and response to entrainment pacing at sites with LDPs [4] (Figure 1). However, many factors make this approach difficult in real practice. First, many patients do not tolerate VT for sufficient time to perform detailed activation and entrainment mapping. Furthermore, clinical VT may not be inducible at the time of procedure. Not infrequently, mechanical block may be induced during activation mapping, and the clinical VT may become temporarily non-inducible. Last, in many cases entrainment may convert one VT circuit into another. To circumvent these issues, alternative ablation approaches during sinus rhythm or limited tachycardia have been proposed. These include linear ablation within the scar, noncontact mapping, pace mapping to identify the VT exit site, identification of non-excitable scar, VT mapping with external hemodynamic support, limited entrainment mapping, focusing premature ventricular complexes to identify VT exit site and mapping for presystolic or diastolic potentials during sinus rhythm [5-12] (Figure 2). These indirect methods of locating re-entrant circuits are highly relevant in clinical practice as up to 80% of postinfarct VTs can have multiple circuits and/or hemodynamically unstable rhythms warranting alternative approaches [13]. However, these alternative approaches do have lower precision compared to mapping during VT and may end up in delivery of higher number of ablative lesions [14]. In a retrospective nonrandomised study in 47 patients with postinfarct VTs, scar mapping during sinus rhythm or right ventricular apical pacing has been found as successful as activation mapping during VT [15]; however, this is yet to be prospectively evaluated in large randomised trials.

The most promising among these alternative techniques seems to be based on mapping of LDPs during sinus rhythm [11,12]. The surviving myocardial bundles traversing infarct borders into deeper scar have decreased gap junction density and myocardial disarray [1]. These insulated myocardial channels promote slow conduction and act as protected isthmuses for re-entry. During sinus rhythm, this conduction delay results in LDPs which represent the local activation persisting even after the depolarisation of remaining healthy myocardium. The extent of this local delay can be further estimated by QRS-LDP interval, as measured as the timing interval from the onset of surface QRS to the latest component of the LDP electrogram. Hsia et al recently found LDPs in more than 90% of VT isthmus sites, independent of the electrogram voltage characteristics [16]. Although right ventricular pacing has been proposed to uncover LDPs in target sites of ablation during substrate-based mapping [11], the incremental value of this approach in improving the ablation outcome is yet to be verified. An additional concern is the limited specificity of these potentials to identify the isthmuses of the re-entrant circuits, as blind loops or innocent bystander loops may co-exist in the substrate. A long stimulus-QRS interval and excellent pace mapping at sites with LDPs would serve as additional tools that help to increase the specificity of the potentials related to tachycardia isthmus. Of these, pace mapping has a limitation in that the centrifugal activation from the electrode tip may differ from unidirectional conduction through the channels during VT if functional blocks during two scenarios are not identical. A few studies have characterised the spatial relationship between LDPs - the surrogate markers of re-entry during sinus rhythm - and the components of re-entrant VT circuits (entrance, mid-isthmus, exit and outer loop) as defined by entrainment criteria during VT [3,16-19]. Hopefully, these improvements in the understanding of LDPs would strengthen the substrate-based ablation strategies in future. Still, it is logical to believe that the success rate of substrate-based approach is likely to remain lower despite longer ablation lines when VT isthmuses are less well defined.

Historically, the first attempts at surgical therapy of VT were guided by epicardial mapping, and fractionated electrograms and LDPs were described in that era of pre-operative VT mapping itself  [20]. The use of epicardial approach to VT ablation has come a complete circle now, and is being increasingly used in the electrophysiology laboratory. Usefulness of this technique depends on the prevalence and pattern of epicardial location of re-entrant circuits in a given case that vary depending on the substrate. Though lower in prevalence compared to patients with Chaga's disease and arrhythmogenic right ventricular dysplasia [21], VT circuits with predominantly epicardial location can be seen in 10-40% of patients taken for ablation of VT late after myocardial infarction [22-24]. Cesario et al, using high-density endocardial and epicardial mapping of hemodynamically unstable VTs, showed that epicardial scars in patients with ischemic cardiomyopathy tend to be lesser in prevalence, patchier  in distribution and less likely to be transmural compared to those with non-ischemic cardiomyopathy [22]. Furthermore, even within the subgroup of post-infarct VTs, epicardial circuits tend to be more prevalent in patients with inferior wall infarctions without aneurysms [23]. This highlights the importance of optimal selection of patients for epicardial approach.

Despite the difficulties related to pericardial access (especially in postoperative cases), inherent risk of damage to coronary arteries and/or phrenic nerve, problems in mapping due to epicardial fat distribution and debates on ideal energy source (radiofrequency vs. cryoablation), this approach is becoming an important adjunctive to standard endocardial approaches in selected cases. Thus the case described by Makhija et al in this issue of the journal is appropriately timed and adds substantively to other reports that detail epicardial approach and mapping of scar-related VTs [25]. In this case, the authors performed substrate-based epicardial ablation in a post-myocardial infarction scar VT with delineation of electrical scarring and re-entrant circuits by mapping LDPs and performing pace mapping at these areas. Prior endocardial approach to ablation was unsuccessful in this case, and VT induced during epicardial approach was hemodynamically unstable. This article provides additional insights regarding the challenges involved in mapping and ablation of scar-related VTs in a real world scenario. Furthermore, this case report underscores the fact that a 'one size fits all method' is not suitable for VT ablative procedures. Instead, a hybrid method combining an individually tailored substrate analysis and various degrees of voltage mapping, pace mapping, activation mapping and entrainment mapping should result in optimal outcome in majority of cases. As catheter ablation has already become an important adjunct to defibrillator therapy in patients with scar-related VTs, and the majority of these patients do have unmappable VTs, the role of ablative approaches during sinus rhythm is likely to increase in the foreseeable future.

Furthermore, this case report underscores the fact that a 'one size fits all method' is not suitable for VT ablative procedures. Instead, a hybrid method combining an individually tailored substrate analysis and various degrees of voltage mapping, pace mapping, activation mapping and entrainment mapping should result in optimal outcome in majority of cases. As catheter ablation has already become an important adjunct to defibrillator therapy in patients with scar-related VTs, and the majority of these patients do have unmappable VTs, the role of ablative approaches during sinus rhythm is likely to increase in the foreseeable future.

## Figures and Tables

**Figure 1:**
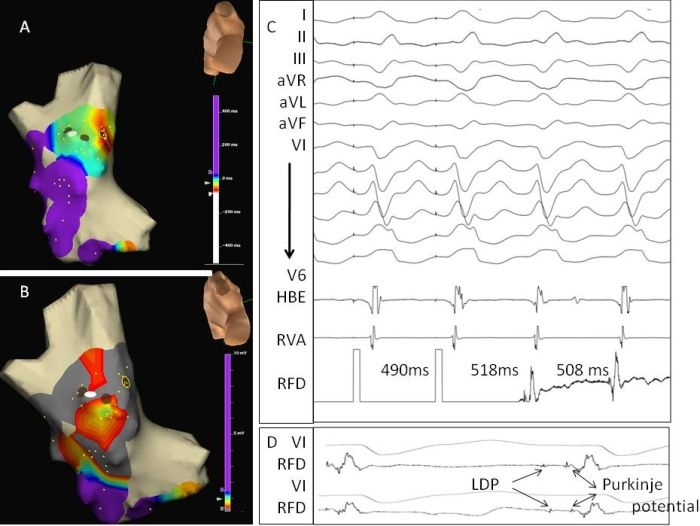
Activation, entrainment and voltage mapping during VT (left bundle branch block morphology and left axis deviation) in a patient with prior inferior wall myocardial infarction. VT had to be terminated intermittently due to borderline hemodynamic status; hence the limited activation map. The brown circles in 1A and 1B represent the sites with LDPs during VT, which have been further characterised by entrainment mapping. All these sites with LDPs showed Purkinje potentials also. Out of these, the brown circle with yellow halo was the closest to the VT exit.  All the mapped LDPs corresponded to the exit site of the re-entrant isthmus (S-QRS/ VT cycle length < 30%, concealed fusion, and entrainment response < 30 ms) by the classification of postinfarction VT re-entrant circuits proposed by Stevenson et al (3) (C and D). The earliest ablation was given at the site with best entrainment response (represented by the white circle), which was about 1.5 cm posterior to the site of earliest activation obtained at limited activation mapping. RF ablation at this point terminated the tachycardia within 20 seconds, and made it noninducible. On voltage map (1B), the red zones near to the site of ablation had bipolar voltage between 0.4 and 0.5mV. Areas in brown display dense scar (≤ 0.4 mV).

**Figure 2:**
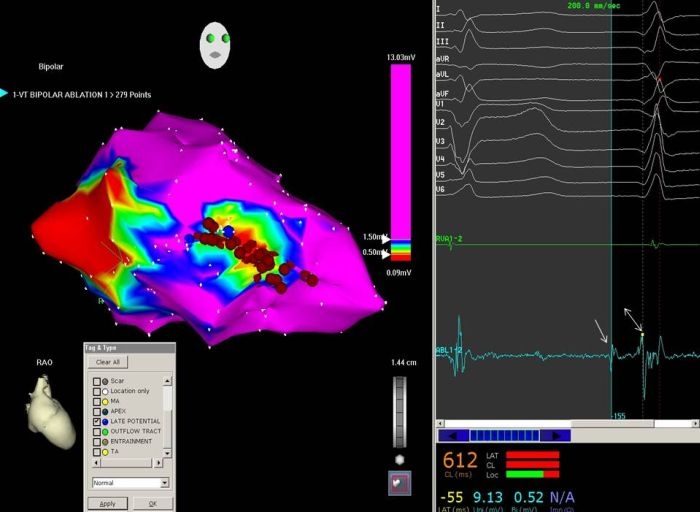
Voltage map of left ventricle in RAO view in an elderly male with previous anterior wall myocardial infarction, left bundle branch block and recurrent ventricular tachycardia. Local bipolar electrogram voltage of ≤  0.5 mV has been arbitrarily selected as the threshold of dense scar (red in colour), ≥ 1.5mV as normal (purple in colour) and 0.5-1.5 mV the borderline zone. The electrogram window shows a sinus beat followed by the initial beat of ventricular tachycardia. The local electrogram from the area marked with blue circle at the scar border zone shows LDPs and Purkinje potential in the diastolic interval preceding the initiation of VT (The potentials marked with arrow and double arrow respectively). Linear ablation as shown by brown circles in the figure, at LDP sites and across the scar, made the tachycardia non-inducible.
